# Preliminary Study of Morphological Variability in the Circle of Willis and Its Clinical Significance in Ethiopia: A Cadaveric Study

**DOI:** 10.4314/ejhs.v35i3.6

**Published:** 2025-05

**Authors:** Guta Bulcha, Asegedech Bekele, Abebe Muche

**Affiliations:** 1 Departmen of Human Anatomy, School of Medicine College of Medicine and Health Sciences, University of Gondar, Gondar, Ethiopia; 2 Department of Human Anatomy, Adama Science and Technology University. Adama, Ethiopia; 3 Department of Human Anatomy, Biomedical Science Division, School of Medicine, University of Global Health Equity, Kigali, Rwanda

**Keywords:** Circle of Willis, anterior cerebral artery, posterior cerebral artery, anterior communicating artery, posterior communicating artery

## Abstract

**Background:**

The Circle of Willis (COW) is a vital cerebral arterial network that maintains balanced blood flow in the brain. Despite initial insights from Thomas Willis, recent research has highlighted its complex anatomical variations.

**Objective:**

This study aims to evaluate the anatomical variations of the COW in Ethiopian cadavers.

**Methods:**

We employed a descriptive observational approach on thirty-two cadavers. Detailed morphological examinations of the COW were performed following meticulous dissection at various Ethiopian universities.

**Results:**

Significant anatomical variations were observed, particularly in the anterior divisions of the COW, with 57.14% of cases exhibiting notable differences. Variations in the anterior communicating artery (62.5%) and anterior cerebral artery (37.5%) were particularly diverse. In the posterior segment, 66.6% of anomalies involved unilateral right hypoplastic posterior communicating arteries, and bilateral hypoplastic posterior cerebral arteries were noted in two male cadavers. Essential vessels were present in all specimens.

**Conclusion:**

Comparative analysis with global studies reveals both similarities and differences, highlighting genetic and methodological influences. Recognizing these variations is crucial for diagnosing cerebrovascular diseases and managing intracranial aneurysms. Further longitudinal studies using advanced imaging techniques are recommended to enhance understanding in the Ethiopian population and beyond.

## Introduction

Thomas Willis (1621–1675) first depicted the Circle of Willis (COW) in his 1664 work *Cerebri Anatome*, though his illustrations did not fully capture the communicating arteries. Vesling (1598–1649) later provided a more complete representation of the posterior communicating arteries but did not definitively show the union of the anterior cerebralarteries (1,2). The comprehensive understanding of the COW, including its anastomotic properties, was achieved through Willis' research (1,2).

The COW, also known as the cerebral arterial circle, is an arterial polygon formed by the anastomosis of the internal carotid and vertebral systems around the optic chiasm and pituitary stalk in the suprasellar cistern (3). It consists of two divisions: the anterior division, comprising the ACA and the anterior communicating artery (AComA), and the posterior division, including the PCA, terminal branches of the basilar artery, and the posterior communicating arteries (PcomA), which connect the internal carotid arteries (ICA) with the PCA (4).

Over the past sixty years, various anatomical variations of the COW have been documented, including absent or aplastic arteries, fenestrated arteries, hypoplastic arteries, duplicated arteries, and accessory vessels (5-12). Understanding these variations is crucial, as the COW facilitates collateral circulation and pressure equalization in the brain. Variations can affect blood flow, potentially increasing the risk of cerebrovascular diseases (13,14). The prevalence of COW variants is higher in stroke patients and recognizing these variations aids in understanding cerebral ischemic disease (15,16). Additionally, the COW's arterial pulsations contribute to cerebrospinal fluid drainage in the interpeduncular cistern (12).

Research on COW variations has been diverse, but generalizing findings to different populations can be challenging (17). The influence of genetic and developmental factors on COW variability is significant. However, the spectrum of COW anatomy in Ethiopia remains largely unexplored. This study aims to examine COW anatomical variations in Ethiopian cadavers.

## Materials and Methods

**Study design**: A descriptive observational study was conducted to assess the morphological variations of the COW in Ethiopian cadavers.

**Study area**: The study was conducted at the University of Gondar (747 km from Addis Ababa), Bahir Dar University College of Medicine and Health Science (565 km), and Adama Science and Technology University (175 km). Fourteen specimens were examined: 10 from the University of Gondar, 2 from Bahir Dar, and 2 from Adama Science and Technology University.

**Study population**: The study included cadavers available for teaching purposes at the above-mentioned universities during the study period.

**The following operational definitions are used**

- **Normal architecture of COW**: A symmetrical ring of arteries at the brain's base providing redundant blood flow paths in case of blockages.
- **Variant architecture of COW**: Deviations from the typical structure, including missing, duplicated, or asymmetrically sized arteries, which may affect blood flow compensation.
- **Fenestration**: Division of the arterial lumen into separate channels with distal convergence.
- **Duplication**: Presence of two distinct arteries with separate origins and no distal convergence.

**While** cadavers without trauma, prepared for teaching at the University of Gondar, Bahir Dar University College of Medicine, and Adama Science and Technology University were considered as inclusion criteria; Cadavers with evidence of brain pathology or trauma affecting arterial topography were used as exclusion criteria.

**Sampling method**: Purposive sampling technique was used.

**Data collection procedure and materials**: Two senior anatomy technicians received one week of training on dissection techniques. After a pre-assessment, they proceeded with dissection. A detailed examination of 32 formalin-preserved cadaver brains was conducted. The dissection involved careful removal of the brain and identification of the COW, with documentation of anatomical variations such as hypoplasia, aplasia, duplication, and fenestrations. Observations were recorded, and photographs were taken for comprehensive analysis.

**Data analysis procedure**: Data was checked for completeness, accuracy, and clarity. Analysis was performed manually using a scientific calculator, with results presented in figures, tables, and percentages.

**Ethical considerations**: Ethical clearance was obtained from the Research and Publication Office of the University of Gondar. Official letters were submitted to the university hospitals, and the study's purpose and importance were explained.

## Results

**Figure 1:**
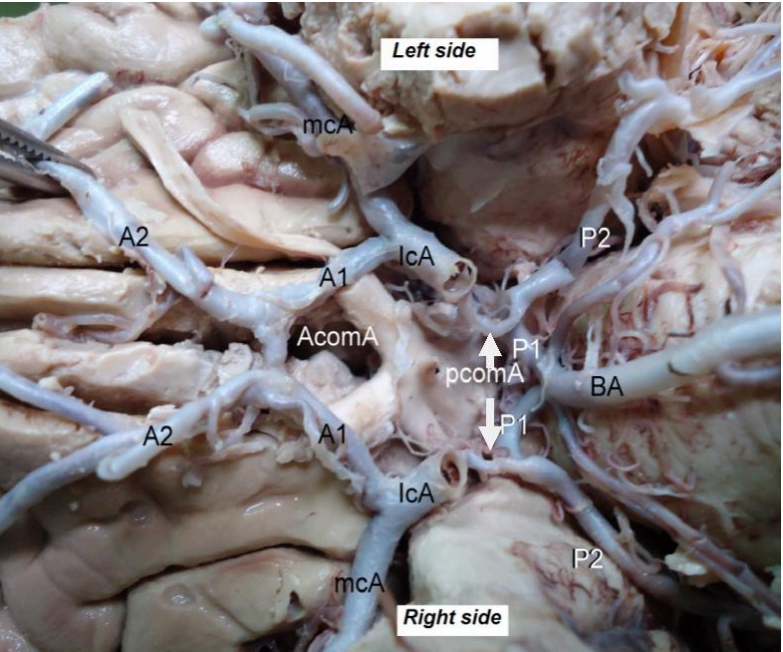
Photograph of a typical adult Circle of Willis from selected specimens

This study analyzed 32 cadavers, consisting of 23 males and 9 females, aged between 32 and 70 years. We thoroughly examined and photographed the entire COW structure, assessing its typicality, completeness, symmetry, and associated blood vessels. Among the 32 cadavers, 18 (56.25%) exhibited a typical COW architecture **(Fig. 1)**, while 14 (43.75%) showed variations.

Variations were most frequently observed in the anterior half of the COW, accounting for 57.14% of cases, with the anterior communicating artery (AComA) responsible for 62.5% of these variations. Posterior variations comprised 42.86% of the total, with the posterior communicating artery (PComA) representing 66.7% of these anomalies.

**Figure 2:**
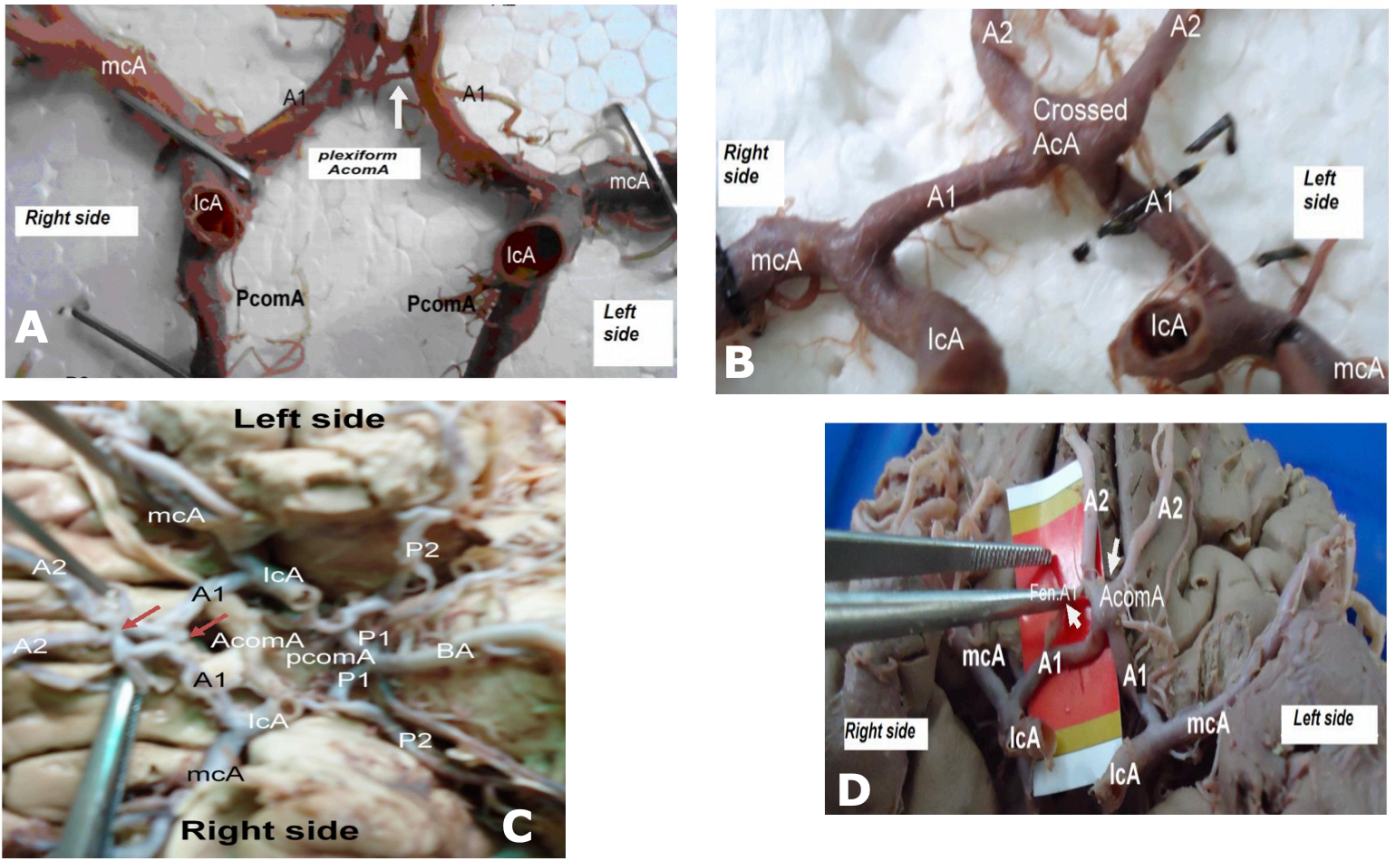
Figure showing (A) a duplicated plexiform AComA (indicated with a white arrowhead), (B) crossed ACA from selected specimens, (C) a double AComA (indicated with a red arrowhead) and (D) A1 fenestrations and fusion of the ACA (indicated with a white arrowhead)

We found that most variations in the COW were concentrated in the anterior half, particularly involving the AComA, which contributed to 62.5% of the observed variations. The most common anomaly was a duplicated plexiform AComA, seen in 4 cases (50% of anterior variations; see **Table 1** and **Fig. 2A, B, C and D**). Crossed ACA (25%) and double AComA (12.5%) were also noted.

**Table 1:** Frequency and percentage of arterial variations in the Circle of Willis

Variant Artery	Male	Female	Total
Double AComA	1 (11.11%)	0 (0%)	1 (7.14%)
Duplicated Plexiform AComA	3 (33.33%)	1 (20%)	4 (28.57%)
Crossed ACA	0 (0%)	2 (40%)	2 (14.29%)
Fenestration and Fused ACA	1 (11.11%)	0 (0%)	1 (7.14%)
Hypoplastic Right PComA	2 (22.22%)	2 (40%)	4 (35.71%)
Hypoplastic PCA	2 (22.22%)	0 (0%)	2 (14.29%)
**Total**	9 (100%)	5 (100%)	14 (100%)

**Figure 3:**
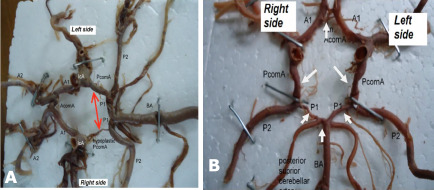
Figure representing (A) a hypoplastic right PComA (indicated with double headed arrow) and (B) bilateral fetal-type PComA and variations in posterior cerebral arteries (indicated with white arrowheads)

For the posterior COW variations, unilateral right hypoplastic PComA was observed in two males **(Fig. 3A)** and two females (66.7% of posterior anomalies). Bilateral hypoplastic PCA was noted in two male cadavers **(Fig. 3B)**.

Despite these variations, no essential component vessels were entirely absent in any of the specimens.

## Discussion

Despite constituting only 2% of the body's mass, the brain consumes a significant portion of cardiac output and oxygen, making it highly sensitive to disruptions in blood supply (18). The COW plays a crucial role in ensuring a stable blood supply to the brain, as first described by Thomas Willis in 1662 (19). Variations in the COW, often influenced by genetic factors, can involve the loss or persistence of vessels or the formation of new vessels due to hemodynamic factors (3,5,17).

While these anatomical variations generally do not significantly affect brain function due to compensatory collateral circulation (20,21), they are important in clinical settings. The COW provides a critical collateral flow to affected brain regions in cases of arterial insufficiency and is significant in preventing ischemia (22,23). Variations in the COW are also associated with a higher prevalence of intracranial aneurysms, particularly at junctions such as the ICA-PComA or AComA (23).

Our study found a notable prevalence of variations in the anterior segments of the COW (57.14%), with significant involvement of the AComA (62.5%). In contrast, posterior anomalies were dominated by unilateral right hypoplastic PComA (66.7%). These findings underscore the importance of understanding COW variations in clinical practice.

Comparing with previous studies, our findings align with Gautam et al. (17), who reported a typical COW in over 50% of specimens. However, other studies have reported lower prevalence rates for typical COWs, which may be influenced by geographical or methodological differences **(see, Table 2)**. Our study shows a higher incidence of anterior segment anomalies compared to some regional studies, highlighting the variability in COW anatomy.

**Table 2:** Comparison of anterior and posterior divisions of the Circle of Willis among various studies

Study	Total	Typical	Anterior Division	Posterior Division	Anterior & Posterior Division
Riggs and Rupp, (11)	994	192(19.3%)	129 (13%)	320 (32.2%)	353 (35.5%)
El Khamlichi et al., (7)	100	18 (18%)	13 (13%)	42 (42%)	27 (27%)
Fisher, (8)	414	20 (4.8%)	6 (1.4%)	221 (53.4%)	167 (40.3%)
Lazorthes et al., (9)	200	29 (14.5%)	12 (6%)	86 (43%)	73 (36.5%)
Eftekhar et al., (6)	102	29 (28.4%)	0 (0%)	51 (50%)	12 (11.8%)
De Silva et al., (5)	225	32 (14.2%)	38 (16.9%)	82 (36.4%)	73 (32.4%)
Klimek Piotrowska et al., (24)	100	27 (27%)	2 (2%)	30 (30%)	41 (41%)
Ciliers et al., (25)	39	16 (41%)	3 (8%)	13 (33%)	7 (18%)
Gautam et al., (17)	107	92 (86%)	9 (8.4%)	5 (4.7%)	1 (0.9%)
Present Study	32	18 (56%)	7 (21.9%)	5 (15.6%)	1 (3.1%)

**Anterior communicating artery (AComA)**: We observed duplicated plexiform AComA in 28.57% of cases and double AComA in 7.1%. Historical studies have documented variations in AComA, with duplication reported in up to 7.2% of cases (26). Other studies report varying incidences of AComA hypoplasia, from 14% to 0% (5-9, 11).

**Anterior cerebral artery (ACA)**: Anatomical variations in the ACA were observed in 37.5% of cases, including configurations such as crossed and fused ACAs. Previous studies have reported similar variations, including the fusion of ACAs into a single azygous artery, which is common in animals (10, 28-31). Our study's incidence of hypoplastic A1 segments (7.14%) is higher compared to some studies (6-9, 11)

In conclusion, our study highlights the anatomical variations within the COW, particularly within its anterior division. These variations often involve combinations of different formative vessels, especially the communicating arteries, which are essential for facilitating collateral circulation and reducing functional impairment. Recognizing these variations is critical in clinical practice, especially concerning cerebrovascular diseases and intracranial aneurysms. We recommend a comprehensive longitudinal study of COW variations within the Ethiopian population using advanced imaging techniques. Clinicians must remain vigilant for anomalies in key arteries. Additionally, collaboration among anatomists, clinicians, and researchers is crucial to advance diagnostic and therapeutic approaches, ultimately improving patient care.
